# Identification and immune features of cuproptosis-related molecular clusters in polycystic ovary syndrome

**DOI:** 10.1038/s41598-022-27326-0

**Published:** 2023-01-18

**Authors:** Zhe Su, Wenjing Su, Chenglong Li, Peihui Ding, Yanlin Wang

**Affiliations:** 1 Department of Reproductive Medicine Center, Binzhou Medical University Hospital, No. 661 Huanghe 2nd Road, Binzhou, 256603 China; 2 Department of Radiology, Binzhou Medical University Hospital, Binzhou, 256603 China; 3 Department of Neurosurgery, Binzhou Medical University Hospital, Binzhou, 256603 China

**Keywords:** Endocrine reproductive disorders, Data acquisition, Data mining, Data processing, Databases, Functional clustering, Machine learning, Microarrays, Statistical methods

## Abstract

Polycystic ovary syndrome (PCOS), a common reproductive endocrine disease, has clinically heterogeneous characteristics. Recently, cuproptosis causes several diseases by killing cells. Hence, we aimed to explore cuproptosis-related molecular clusters in PCOS and construct a prediction model. Based on the GSE5090, GSE43264, GSE98421, and GSE124226 datasets, an analysis of cuproptosis regulators and immune features in PCOS was conducted. In 25 cases of PCOS, the molecular clusters of cuproptosis-related genes and the immune cell infiltration associated with PCOS were investigated. Weighted gene co-expression network analysis was used to identify differentially expressed genes within clusters. Next, we compared the performance of the random forest model, support vector machine model, generalized linear model, and eXtreme Gradient Boosting for deciding the optimum machine model. Validation of the predictive effectiveness was accomplished through nomogram, calibration curve, decision curve analysis, and using other two datasets. PCOS and non-PCOS controls differed in the dysregulation of cuproptosis-related genes and the activation of immunoreaction. Two cuproptosis-related molecular clusters associated with PCOS were identified. Significant heterogeneity was noted in immunity between the two clusters based on the analysis of immune infiltration. The immune-related pathways related to cluster-specific differentially expressed genes in Cluster1 were revealed by functional analysis. With a relatively low residual error and root mean square error and a higher area under the curve (1.000), the support vector machine model demonstrated optimal discriminative performance. An ultimate 5-gene-based support vector machine model was noted to perform satisfactorily in the other two validation datasets (area under the curve = 1.000 for both). Moreover, the nomogram, calibration curve, and decision curve analysis showed that PCOS subtypes can be accurately predicted. Our study results helped demonstrate a comprehensive understanding of the complex relationship between cuproptosis and PCOS and establish a promising prediction model for assessing the risk of cuproptosis in patients with PCOS.

## Introduction

Polycystic ovary syndrome (PCOS) is a common reproductive endocrine disorder with a prevalence of 5–10% among women of childbearing age, with its main feature being chronic anovulation^1^. As a reproductive system disease, PCOS causes disorder of sex hormones in the ovaries of women of childbearing age. One of the clinical manifestations of PCOS is an irregular menstrual cycle, resulting in ovulation dysfunction. According to a large community-based cohort study, 72% of women with PCOS were infertile compared with 16% of those without PCOS^2^. Therefore, PCOS significantly aggravates the occurrence of infertility. Owing to its clinical heterogeneity, regrettably, PCOS is a complex disease and patients respond differently^3^. Meanwhile, since the symptoms of patients with PCOS are not unified, the diagnosis of PCOS is quite difficult^4^. Therefore, it is of great clinical importance to further accurately identify PCOS at the molecular levels with its molecular subtypes and create a multivariate predictive model.

As eukaryotic organelles, mitochondria participate in various basic functions in organisms, including iron–sulfur cluster synthesis, copper homeostasis, lipid and amino acid metabolism, and energy transduction^5^. They are also an important site for copper utilization. Meanwhile, several key mitochondria enzymes require copper as a cofactor^6^. However, the findings of a related study suggested that increased copper levels in the follicular fluid may harm follicle development in patients with PCOS. Therefore, the toxicity of copper may have adverse effects on human reproduction^7^. A new mechanism for cell death, cuproptosis, which is different from the known mechanisms, has been recently discovered. Copper triggers a non-apoptotic form of cell death that depends on mitochondrial respiration. The direct binding of copper to the lipoylated components of the tricarboxylic acid cycle leads to abnormal aggregation of lipoylated proteins and loss of iron–sulfur cluster proteins, causing proteotoxic stress and finally cell death^8^. PCOS, a type of metabolic dysfunction, causes systemic disorder^9^. The energy balance of a cell is maintained by aerobic glycolysis and mitochondrial oxidative phosphorylation^10^. Moreover, abnormal mitochondrial function contributes to the progression of metabolic diseases including PCOS. For instance, based on a gene expression analysis of endometrial tissue and skeletal muscle tissue, mitochondrial oxidative metabolism genes expressed lower levels in patients with PCOS than in healthy controls^11^. Furthermore, insulin resistance is a feature of PCOS and is associated with mitochondrial function. The impairment of mitochondrial function is seen in patients with PCOS, which is characterized by reduced oxygen consumption and increased production of reactive oxygen species. There is a hypothetical correlation between insulin resistance and mitochondrial oxidative metabolism impairment in PCOS^12^. Moreover, the central causative factor of PCOS etiology is mitochondria-generated oxidative stress and chronic inflammation^13^. Therefore, we can reasonably conclude that cuproptosis and PCOS development are closely related. However, it remains unknown how cuproptosis is regulated in PCOS. Thus, further research on the molecular features of cuproptosis-related genes (CRGs) may provide insight into PCOS heterogeneity.

In this study, we examined and analyzed the differentially expressed CRGs and immune features between patients with PCOS and healthy controls. Using 15 differentially expressed CRGs as a platform, we categorized 25 patients with PCOS into two clusters related to cuproptosis and compared their immune cells further. Subsequently, weighted gene co-expression network analysis (WGCNA) was used to identify cluster-specific differentially expressed genes (DEGs), which were then used to highlight enriched biological functions and pathways. In addition, through the comparison of multiple machine-learning algorithms, we created a prediction model that was used to reveal patients who have different molecular clusters. Finally, the predictive model was validated for accuracy by nomogram analysis, calibration curve analysis, decision curve analysis (DCA), and other two datasets.

## Methods

### Data selection and pre-processing

The Gene Expression Omnibus (GEO) database (http://www.ncbi.nlm.nih.gov/geo) was searched using the following keywords: (“PCOS” OR “polycystic ovary syndrome” OR “Stein-Leventhal syndrome” OR “sclerocystic ovarian degeneration” OR “sclerocystic ovary syndrome” OR “Sclerocystic Ovary”). The “expression profiling by array” and “homosapiens” filters were applied to search within GEO. In the GEO database, six PCOS-related datasets (GSE5090, GSE43264, GSE98421, GSE124226, GSE80432, and GSE106724) were selected from the search and filter. The GSE5090 dataset (GPL96 platform) included the omental adipose tissue samples from eight healthy controls and nine patients with PCOS; the GSE43264 dataset (GPL15362 platform) included the subcutaneous adipose tissue samples from seven healthy controls and eight patients with PCOS; the GSE98421 dataset (GPL570 platform) included the subcutaneous adipose tissue samples from four healthy controls and four patients with PCOS; and the GSE124226 dataset (GPL570 platform) included the subcutaneous adipose tissue samples from four healthy controls and four patients with PCOS. These four microarray datasets containing a total of the gene expression profiles of 25 patients with PCOS and 23 healthy controls were considered as the train datasets for subsequent investigation. The GSE80432 dataset (GPL6244 platform) included the ovarian granulosa cell samples from eight healthy controls and eight patients with PCOS; the GSE106724 dataset (GPL21096 platform) included the ovarian granulosa cell samples from four healthy controls and eight patients with PCOS. These two datasets were selected for validation analysis. After obtaining the data, we first merged the four datasets (GSE5090, GSE43264, GSE98421, and GSE124226) to increase the sample size and make the experimental results more convincing. Then, a batch correction was conducted on the merged data through the R package "sva" (version 3.44.0) on R Software (version 4.2.1) to remove the batch effect and eliminate potential differences in each dataset^14^.

### Acquisition, differential expression, and correlation analysis of CRGs

Based on previous literature, 16 genes associated with cuproptosis^8,15,16^, including NFE2L2, NLRP3, ATP7B, ATP7A, SLC31A1, FDX1, LIAS, LIPT1, DLD, DLAT, PDHA1, PDHB, MTF1, CDKN2A, DBT and DLST, were collected. A gene analysis was performed using the R package "limma" (version 3.52.4) to compare patients with PCOS with healthy controls^17^. With the Mann–Whitney U test, it was determined whether CRGs were differentially expressed in healthy controls and patients with PCOS, and the differentially expressed CRGs were defined as genes with *P* value < 0.05. Finally, to clarify the interrelationship between the differentially expressed CRGs, a correlation analysis of them was performed.

### Evaluation of immune cell infiltration

The CIBERSORT algorithm and LM22 signature matrix were used in gene expression data to calculate the relative abundance of 22 immune cell types between patients with PCOS and healthy controls^18^. Using Monte Carlo sampling, we calculated deconvoluted *P* value of each sample via the CIBERSORT algorithm, which provided a measure of confidence in the results^19^. Based on the CIBERSORT output of samples with *P* value < 0.05, it could be considered that the fractions of the estimated immune cell population were accurate^20^. The sum of all estimated immune cell type scores in each sample equaled one^21^.

### Correlation analysis between CRGs and infiltrated immune cells

Our analysis of the correlation coefficients between the expression of CRGs and the relative percentage of immune cells provided further evidence for the interrelation between immune cells related to PCOS and CRGs. *P* < 0.05 indicated a significant correlation based on the spearman correlation coefficient.

### Unsupervised clustering of patients with PCOS and principal component analysis (PCA)

Through the k-means algorithm with 50 iterations on the expression of 15 differentially expressed CRGs, 25 patients with PCOS were categorized into different clusters according to unsupervised clustering analysis based on the R package “ConsensusClusterPlus” (version 1.60.0)^22^. The optimal number of clusters was assessed based on a maximum number k (k = 9) of subtypes using a consensus matrix, cumulative distribution function (CDF) curves, and consistent cluster score (> 0.9). Then, the differences between subtypes were further analysed through PCA^23^.

### Gene set variation analysis (GSVA)

For comparing the enriched gene sets between clusters of CRGs, GSVA enrichment analysis was performed using the R package “GSVA” (version 1.44.5)^24^. Further GSVA analysis was performed on the “c2.cp.kegg.v2022.1.Hs.symbols” and “c5.go.v2022.1.Hs.symbols” files downloaded from the Molecular Signatures Database website database (https://www.gsea-msigdb.org/gsea/msigdb). The differentially expressed pathways and biological functions were identified using the R package “limma” (version 3.52.4) by comparing the GSVA scores between different CRGs clusters^17,25^. Significant changes were considered only if the | t value of the GSVA score | was > 2.

### WGCNA

WGCNA was performed to identify co-expression modules using the R package “WGCNA” (version 1.71)^26,27^. According to the variance of the expression amount of each gene in diverse samples in the training dataset, the genes were ranked. To ensure the accuracy of the quality results, the top 25% genes with the highest variance for subsequent WGCNA were selected. A weighted adjacency matrix was constructed using the optimal soft power and then transformed into a topological overlap matrix (TOM). By setting a minimum module size of 100, the TOM dissimilarity measure (1-TOM) was used to obtain modules according to the algorithm of the hierarchical clustering tree. Next, the modules were clustered to observe the similarity among them. A different color was randomly assigned to a distinct module. Module eigengene displayed gene expression profiles for each module. Module significance represented the relationship between modules and disease status. Module membership referred to the correlation coefficient between genes and module eigengenes, which described how reliable a gene in a module was^28^. Gene significance represented the association between gene and clinical traits, with higher gene significance indicating that the specified gene was more relevant to the studied traits. To identify significant modules, the association between modules and clinical traits was calculated and the core genes of this module were output.

### Construction of predictive model based on multiple machine learning methods

The R package "VennDiagram" (version 1.7.3) was used to intersect the key genes between PCOS and its clusters. According to the mutual genes of PCOS with its clusters, the R package “caret” (version 6.0-93) was used to construct machine learning models that contained a random forest (RF) model, support vector machine model (SVM), generalized linear model (GLM), and eXtreme Gradient Boosting (XGB). As a regression tree technique, RF achieves a high degree of predictive accuracy by using bootstrap aggregation and randomization of predictors^29^. SVM is a powerful classification tool that has the function of maximization (support) of separating margin (vector)^30^. GLM, as a generalization of the ordinary linear regression, can examine how categorical or continuous independent characteristics associate with normally distributed dependent characteristics flexibly^31^. XGB is an optimized distributed gradient boosting library; therefore, it is possible to compare classification error and model complexity with great accuracy^32^. These four machine methods were used to identify significant predictive genes of cluster-specific DEGs in PCOS. Different clusters were taken as response variables and cluster-specific DEGs as explanatory variables. A training set (70%, n = 18) and a validation set (30%, n = 7) were randomly divided from the 25 patients with PCOS. All parameters in these models were automatically adjusted using R package “caret” (version 6.0-93) via grid search; fivefold cross-validation was performed to assess each model. The R package “DALEX” (version 2.4.2) was performed to explain the abovementioned four machine learning models and generate the results of residual distribution and feature importance among them^33^. The area under receiver operating characteristic (ROC) curves (AUC) was visualized using the R package “pROC” (version 1.18.0). AUC was calculated to judge the accuracy of the predictive model, and a higher AUC represented a model with higher accuracy^34,35^. Consequently, the optimum machine learning model was determined. Based on the root mean square error, the top five variables of each model were sorted and considered to be important predictive genes related to PCOS. The predictive model was validated for its diagnostic value using ROC curves analysis in the GSE80432 and GSE106724 datasets.

### Construction and validation of a nomogram model

A nomogram model was made for risk assessment of PCOS with the R package “rms” (version 6.3-0). Nomogram had corresponding scores for each key gene; a total score was obtained by combining the scores of five key genes. Therefore, the risk of PCOS could be reflected based on the total score. The predictive ability of nomogram model was assessed using the calibration curve and DCA.

### Independent data validation analysis

The samples from the two ovarian granulosa cell datasets GSE80432 and GSE106724 were used as validation sets to verify the power of the prediction model to discriminate patients with PCOS from healthy controls through the ROC analysis.

## Results

### Dysregulation of CRGs and activation of the immunoreaction in patients with PCOS

**Figure 1:**
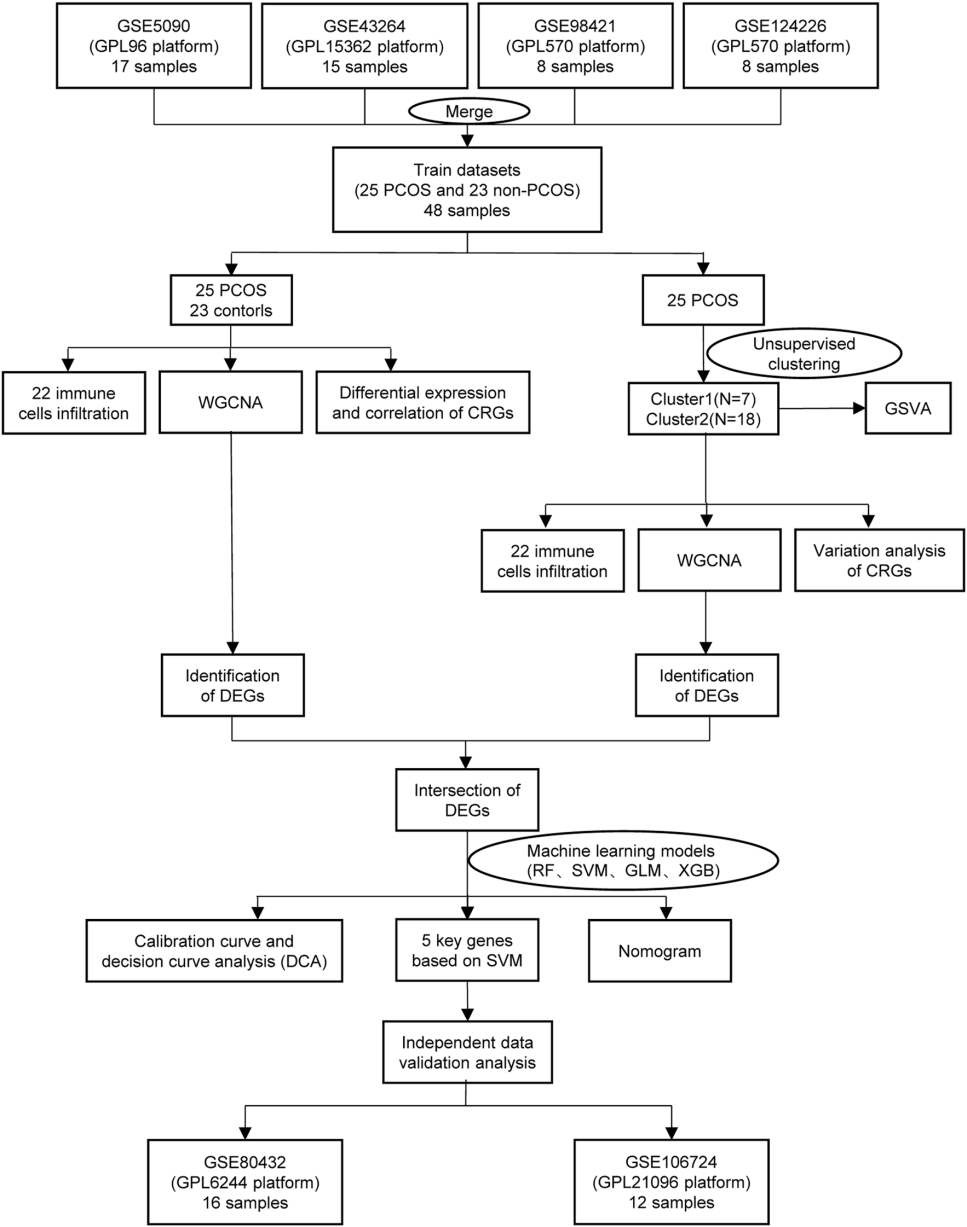
The flow chart of study.

**Figure 2:**
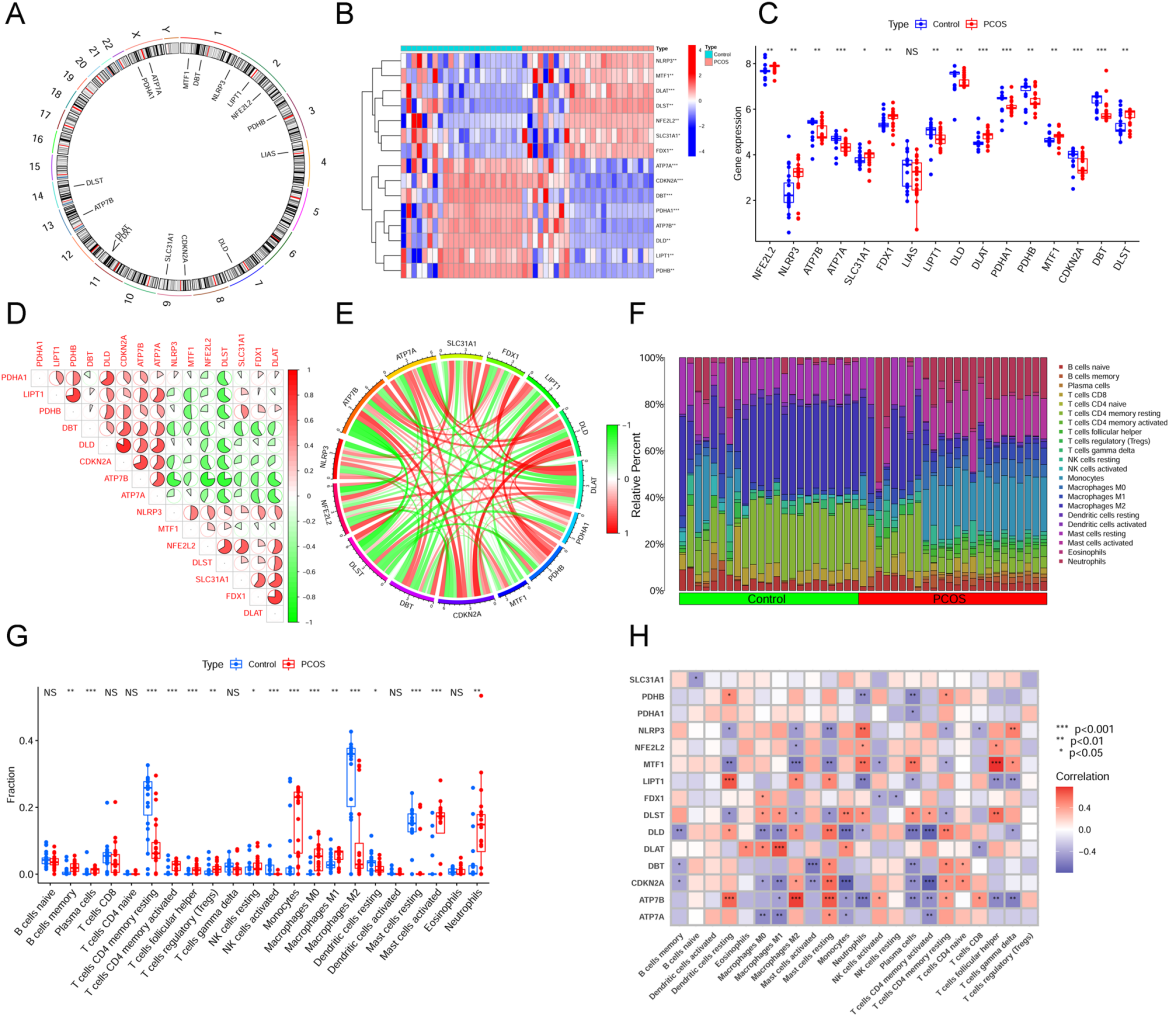
Identification of dysregulated CRGs in PCOS. (**A**) Chromosome location of 16 CRGs. (**B**) Heatmaps showed the expression patterns of 15 differentially expressed CRGs. (**C**) The differential expression of 16 CRGs between PCOS and control group was shown in boxplots. ("***" = *P* < 0.001, "**" = *P* < 0.01, "*" = *P* < 0.05, "NS" = no significance) (**D**) Correlation analysis of 15 differentially expressed CRGs. Colors red and green indicate correlations that are positive and negative, severally. The pie chart area corresponds to the correlation coefficients. (**E**) Gene relationship circos plot of 15 differentially expressed CRGs. The darker the color of the line, the more significant the correlation. (**F**) Bar plot displayed the relative percent of 22 infiltrated immune cells between PCOS and control group. (**G**) The differences in immune infiltrating between PCOS and control group were shown in boxplots. ("***" = *P* < 0.001, "**" = *P* < 0.01, "*" = *P* < 0.05, "NS" = no significance) (**H**) Correlation analysis between 15 differentially expressed CRGs and infiltrated immune cells.

To explore whether CRGs were associated with biological functions in the occurrence of PCOS, the expression profiles of 16 CRGs were comprehensively assessed between patients with PCOS and healthy controls using four datasets from the training group, which were GSE5090, GSE43264, GSE98421, and GSE124226. Figure 1 showed the specific flow chart of the research. A total of 15 CRGs were differentially expressed genes of cuproptosis. We found that the expression levels of DLAT, NLRP3, MTF1, DLST, NFE2L2, FDX1, and SLC31A1 were higher in patients with PCOS than in healthy controls, whereas the gene expression levels of ATP7A, CDKN2A, DBT, PDHA1, ATP7B, DLD, LIPT1, and PDHB were lower in patients with PCOS than in healthy controls (Fig. 2A–C). Afterward, correlation analysis was conducted among these differentially expressed CRGs to determine whether they played an important role in the progression of PCOS. Surprisingly, we found a significant synergistic effect of some CRGs, such as CDKN2A and DLD (coefficient = 0.82). Inversely, ATP7B and DLST presented a strong antagonistic action (coefficient = − 0.77). Furthermore, we found that both CDKN2A and ATP7B in these CRGs were prominently correlated with other genes through further observation (Fig. 2D). The gene relationship circos plot further certified the close association among these differentially expressed CRGs (Fig. 2E).

To clarify whether patients with PCOS and healthy controls had different immune systems, we conducted an immune infiltration analysis and revealed the difference in the proportion of 22 kinds of infiltrating immune cells between patients with PCOS and healthy controls using the CIBERSORT algorithm (Fig. 2F). The results showed that patients with PCOS had higher proportions of activated mast cells, M0 macrophages, monocytes, activated memory CD4^+^ T cells, follicular helper T cells, plasma cells, memory B cells, regulatory T cells (Tregs), M1 macrophages, neutrophils, and resting NK cells (Fig. 2G), indicating that PCOS might have been caused by changes in the immune system. Simultaneously, based on correlation analysis, both follicular helper T cells and activated memory CD4^+^ T cells were also associated with differentially expressed CRGs (Fig. 2H). Based on these results, CRGs might be critical factors in regulating the immune infiltration of patients with PCOS.

### Identification of cuproptosis clusters in PCOS

**Figure 3:**
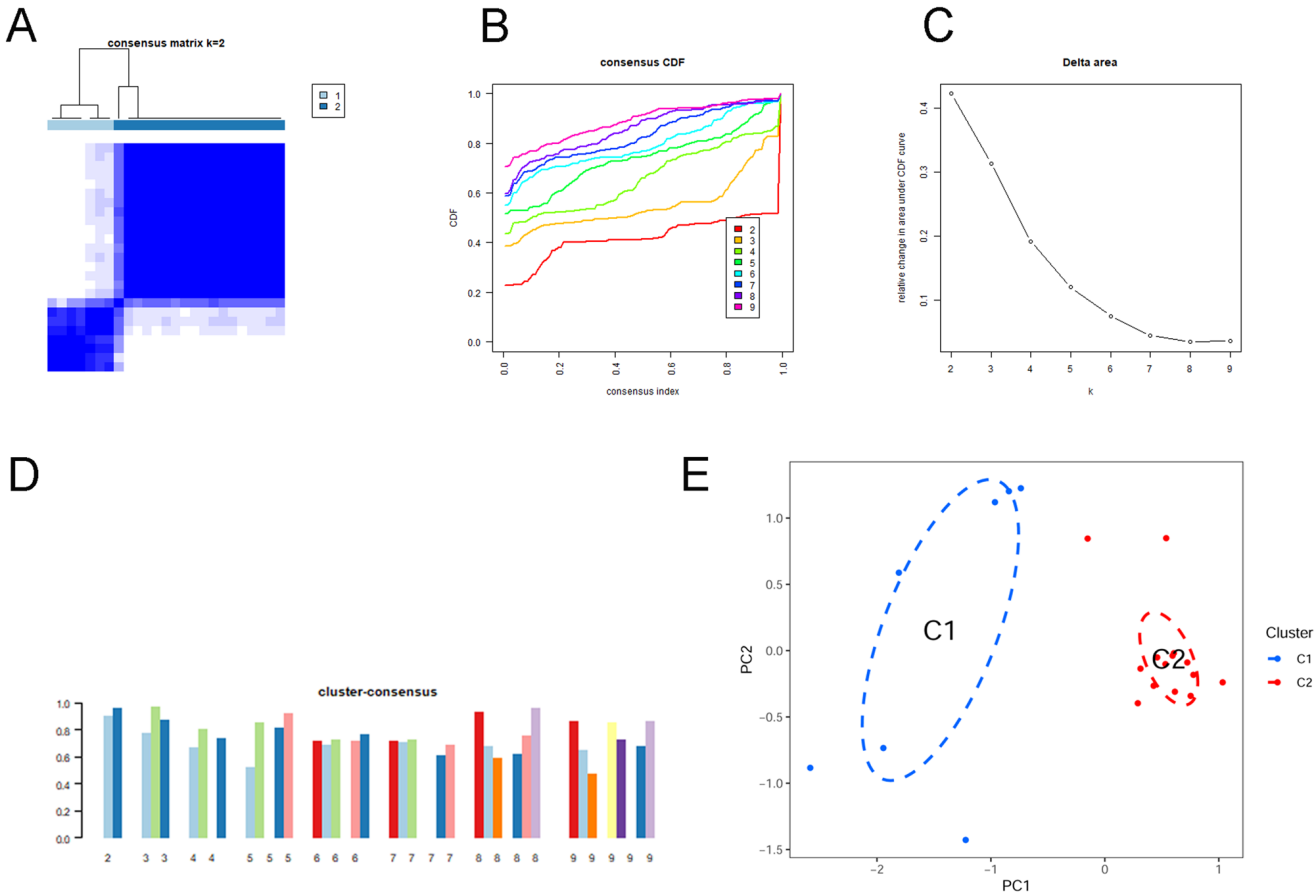
Identification of molecular clusters related to cuproptosis in PCOS. (**A**) Cluster-consensus matrix when k = 2. (**B**) Cumulative distribution function (CDF) curves. (**C**) CDF delta area curves. (**D**) The score of cluster-consensus. (**E**) PCA visualized the distribution of two subtypes.

**Figure 4:**
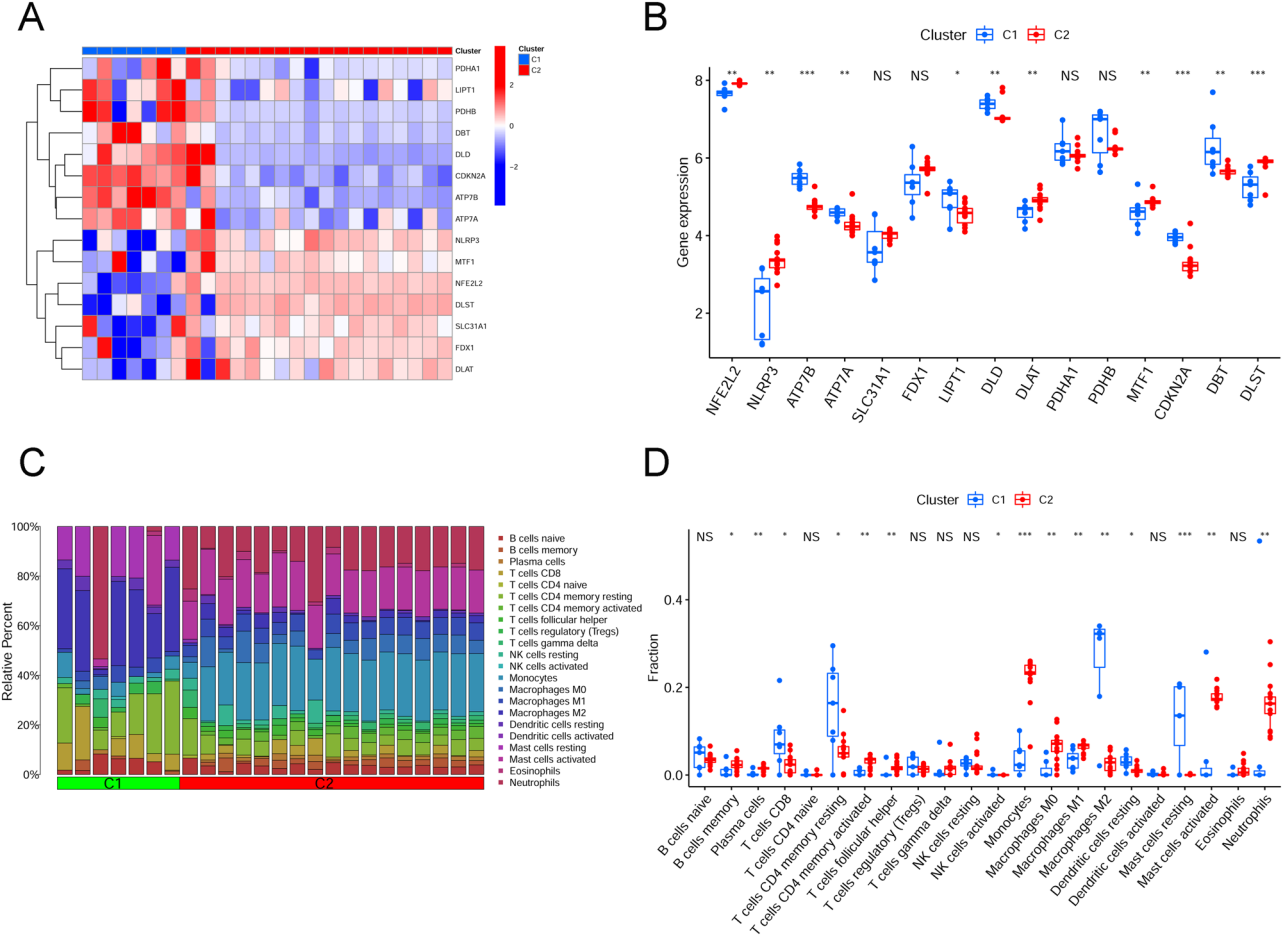
Identification of CRGs and immune features between two cuproptosis clusters. (**A**) Heatmaps showed the expression patterns of 15 differentially expressed CRGs between two cuproptosis clusters. (**B**) The differential expression of 15 differentially expressed CRGs between two cuproptosis clusters were presented in boxplots. ("***" = *P* < 0.001, "**" = *P* < 0.01, "*" = *P* < 0.05, "NS" = no significance) (**C**) Bar plot displayed the relative percent of 22 infiltrated immune cells between two cuproptosis clusters. (**D**) The differences in immune infiltrating between two cuproptosis clusters were presented in boxplots. ("***" = *P* < 0.001, "**" = *P* < 0.01, "*" = *P* < 0.05, "NS" = no significance).

A consensus clustering algorithm was used to group 25 patients with PCOS based on the expression profiles of 15 differentially expressed CRGs to show the expression patterns associated with cuproptosis. When k = 2, the cluster numbers were most steady and the results of cluster analysis were most reliable; the CDF curve fluctuated within the minimum range of the consensus index (0.2–0.6) (Fig. 3A, B). As k = 2–9, the area under the CDF curve differed from k − 1 relative to k (Fig. 3C). Furthermore, only when k = 2, each subtype had a consistency score of > 0.9 (Fig. 3D). After analyzing the consensus matrix heatmap, we ultimately divided 25 patients with PCOS into two clusters, Cluster1 (n = 7) and Cluster2 (n = 18) (Fig. 4A). Meanwhile, there was a significant variation between these two clusters as determined by PCA (Fig. 3E).

### Differentiation of CRGs and immune infiltration features between cuproptosis clusters

Initially, 15 differentially expressed CRGs were systematically compared between Cluster1 and Cluster2 to determine how they differed in expression and to analyze molecular characteristics between clusters. The expression patterns of CRGs were discrepant in the different clusters (Fig. 4A). Cuproptosis Cluster1 showed high expression levels of LIPT1, DBT, DLD, CDKN2A, ATP7B, and ATP7A, whereas the cuproptosis Cluster2 showed high expression levels of NLRP3, MTF1, NFE2L2, DLST, and DLAT (Fig. 4B). Furthermore, immune infiltration analysis revealed changes in the immune system between Cluster1 and Cluster2 (Fig. 4C). Cluster1 showed greater infiltration levels of CD8^+^ T cells, resting memory CD4^+^ T cells, activated NK cells, M2 macrophages, resting dendritic cells, and resting mast cells. However, the abundance of memory B cells, plasma cells, activated memory CD4^+^ T cells, follicular helper T cells, monocytes, M0 macrophages, M1 macrophages, activated mast cells, and neutrophils were comparatively higher in Cluster2 (Fig. 4D).

### Gene modules screening and co-expression network construction

**Figure 5:**
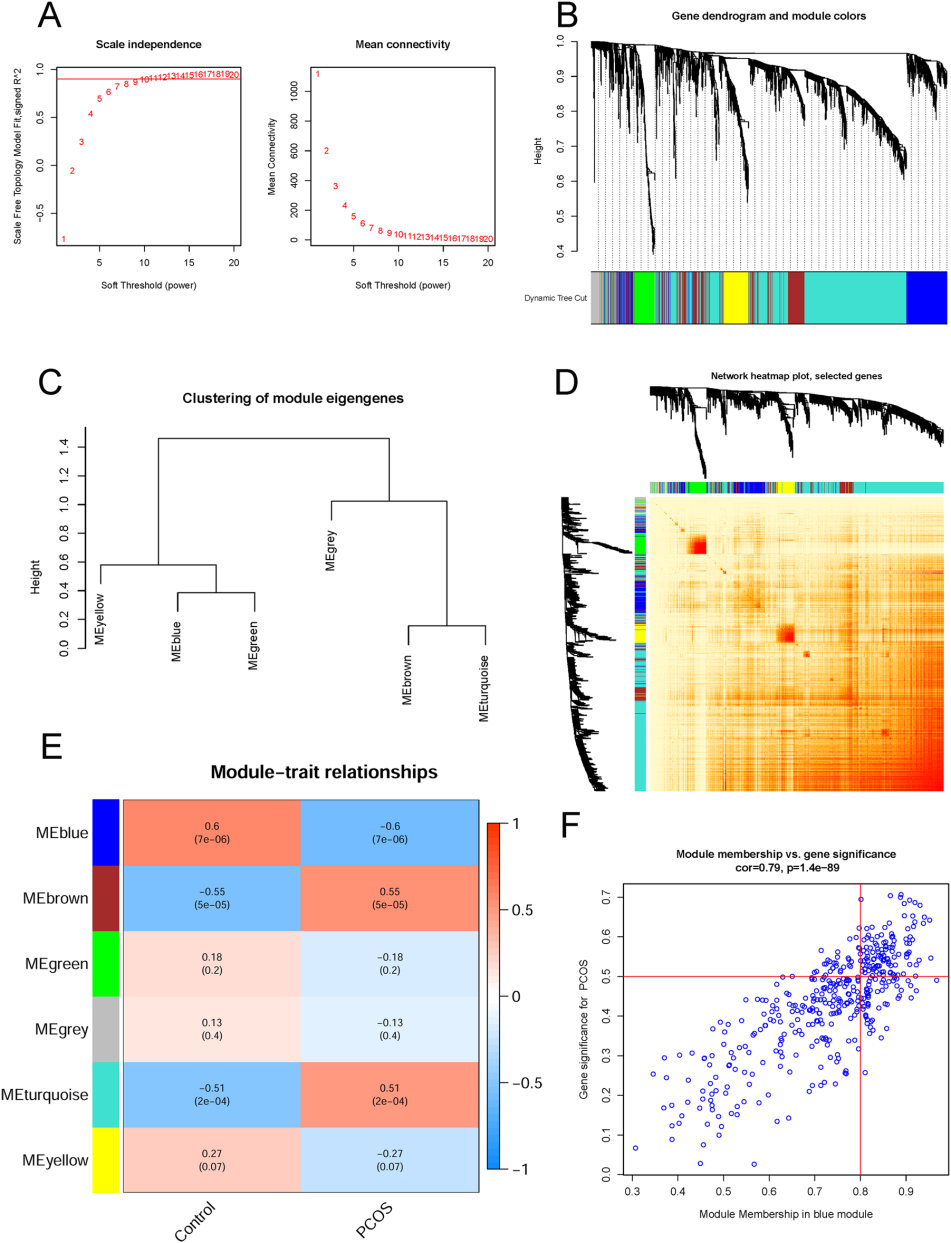
Co-expression network of DEGs in PCOS. (**A**) The option of soft threshold (power). (**B**) Clustering dendrogram of genes in co-expression modules. Distinct co-expression modules were shown in different colors. (**C**) Clustering of module eigengenes. (**D**) The correlations heatmap among 6 modules. (**E**) Correlation analysis between module eigengenes and clinical status. A module is represented by a row, and a clinical status is represented by a column. (**F**) Scatter plot between module membership in blue module and the gene significance for PCOS.

We were able to create co-expression networks and modules for PCOS and healthy controls using the WGCNA algorithm to identify the key gene modules concerned with PCOS. A computation of variance was performed on each gene expression in the training datasets; then, further analysis was conducted on the top 25% of genes with the highest variance. The co-expressed gene modules were identified when the soft threshold was set to 9 and scale-free R^2^ was equal to 0.9 (Fig. 5A). The dynamic cutting algorithm resulted in six distinct co-expression modules with different colors; the heatmap of TOM was also provided (Fig. 5B–D). To assess the resemblance and adjacency of module-clinical traits (PCOS and Control) co-expression, these genes within six modules were analyzed. Finally, PCOS was most strongly associated with the blue module including 142 genes (Fig. 5E). Furthermore, the blue module was positively associated with module-related genes (Fig. 5F).

**Figure 6:**
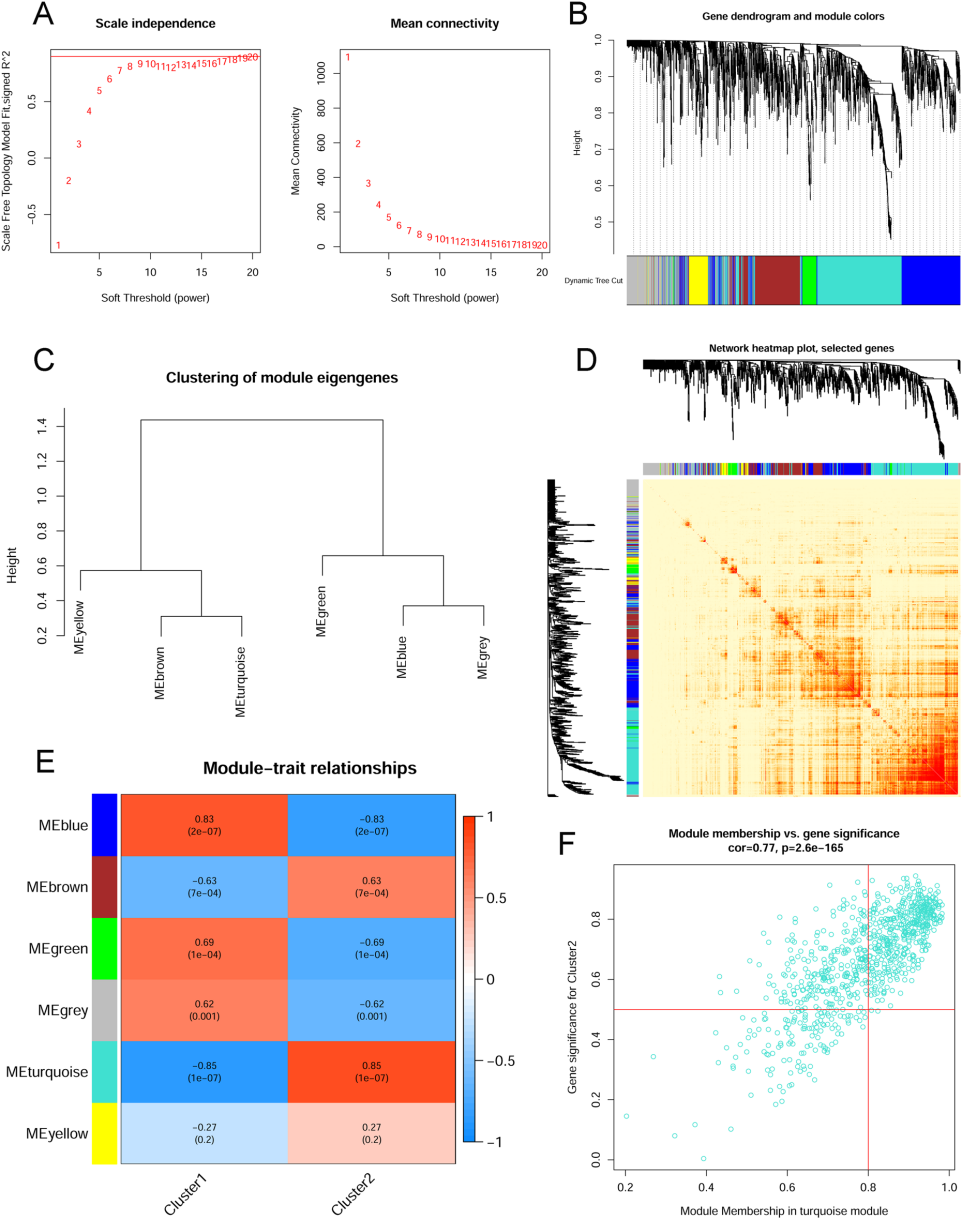
Co-expression network of DEGs between two cuproptosis clusters. (**A**) The option of soft threshold (power). (**B**) Clustering dendrogram of genes in co-expression modules. Distinct co-expression modules were shown in different colors. (**C**) Clustering of module eigengenes. (**D**) The correlations heatmap among 6 modules. (**E**) Correlation analysis between module eigengenes and clinical status. A module is represented by a row, and a clinical status is represented by a column. (**F**) Scatter plot between module membership in turquoise module and the gene significance for Cluster2.

Moreover, using the WGCNA algorithm, the key gene modules closely associated with cuproptosis clusters were expounded. Following the confirmation of the optimal soft threshold parameters (soft threshold = 17, R^2^ = 0.9), the scale-free network was constructed (Fig. 6A). Six significant modules including 2647 genes were finally determined; TOM associated with modules was shown as a result of the heatmap (Fig. 6B–D). According to the module-clinical traits (Cluster1 and Cluster2) relationship explanation, the PCOS clusters were highly associated with the turquoise module (569 genes) (Fig. 6E). Furthermore, the turquoise module genes were strongly associated with the selected module when a correlation analysis was conducted (Fig. 6F).

### Identification of cluster-specific DEGs and biological characteristics

**Figure 7:**
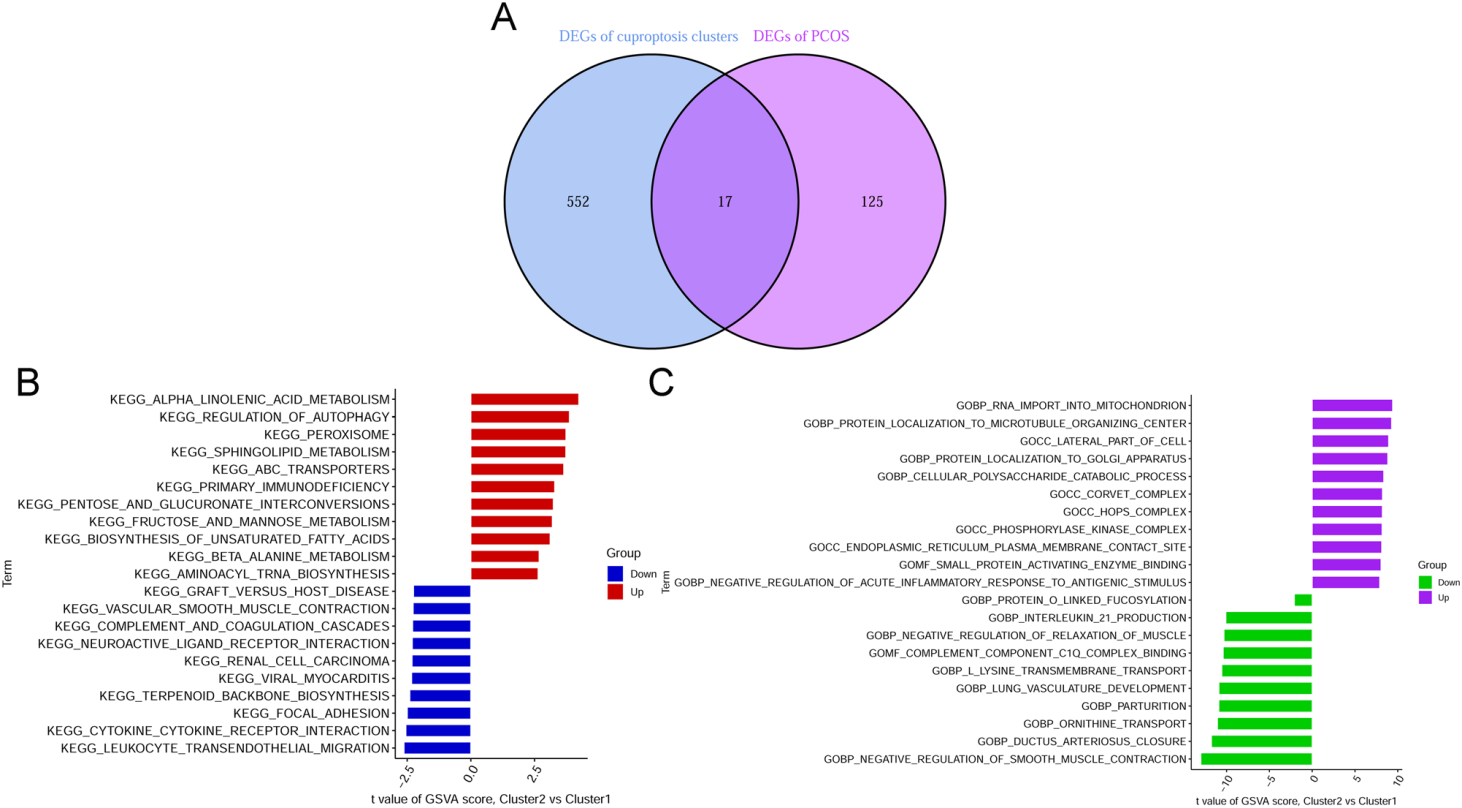
Identification of cluster-specific DEGs and biological characteristics between two cuproptosis clusters. (**A**) The intersections between genes related to module in cuproptosis clusters and genes related to module in GSE5090, GSE43264, GSE98421, and GSE124226 datasets. (**B**) Differences in hallmark pathway activities between Cluster1 and Cluster2 samples were sorted by t-value of GSVA method. (**C**) Differences in biological functions between Cluster1 and Cluster2 samples were sorted by t-value of GSVA method.

By analyzing the intersections of genes related to modules in cuproptosis clusters and PCOS, 17 cluster-specific DEGs were identified (Fig. 7A). GSVA analysis was further used to investigate whether the cluster-specific DEGs in the two clusters had pathway differences. The results suggested that the leukocyte transendothelial migration, cytokine–cytokine receptor interaction, focal adhesion, complement and coagulation cascades, and graft versus host disease were upregulated in Cluster1, whereas the metabolism of alpha-linolenic acid, regulation of autophagy, peroxisome, and sphingolipid metabolism were enriched in Cluster2 (Fig. 7B). Furthermore, the results of functional enrichment indicated that Cluster1 was prominently associated with immune-related pathways, such as interleukin-21 production, and complex binding of complement component C1q, whereas, in Cluster2, there was a reinforcement of the import of RNA into mitochondria, localization of proteins to the Golgi apparatus, lateral part of the cell, protein localization to microtubule organizing center, and cellular polysaccharide catabolic process (Fig. 7C). Therefore, we hypothesized that Cluster1 plays a role in various immune responses.

### Construction and assessment of machine learning models

**Figure 8:**
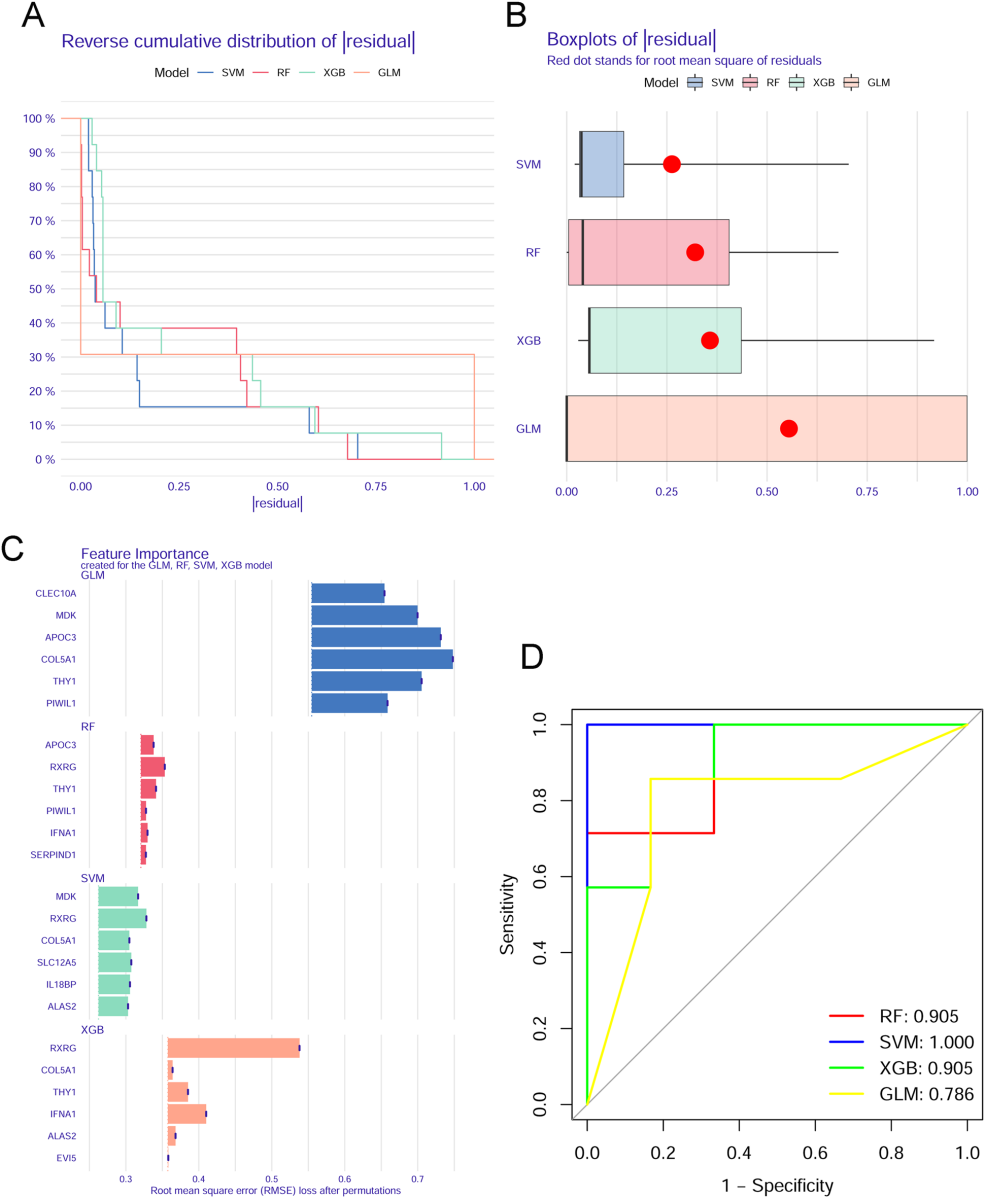
Construction and assessment of RF, SVM, GLM, and XGB machine learning models. (**A**) The reverse cumulative residual distribution of each machine learning model. (**B**) The residuals of each machine learning model were presented in boxplots. The root mean square of residuals is shown as a red dot. (**C**) The important features in four machine models. (**D**) ROC analysis of four machine models based on fivefold cross-validation in the testing group.

According to the expression profiles of 17 cluster-specific DEGs in the PCOS training group, we built four validated machine-learning models (RF, SVM, GLM, and XGB) to identify cluster-specific genes with high diagnostic values. A relatively lower residual was found for the SVM and RF machine learning models (Fig. 8A,B). The feature importance diagram of each machine learning model based on root mean square error was provided (Fig. 8C). Furthermore, to measure discriminative performance of each machine learning algorithm in the testing group, we calculated ROC curves via fivefold cross-validation. The highest AUC was observed in the SVM machine learning model (AUC_RF_ = 0.905; AUC_SVM_ = 1.000; AUC_XGB_ = 0.905; AUC_GLM_ = 0.786; Fig. 8D). On the whole, the SVM model was proven to be the optimum model for identifying patients with PCOS from different clusters combined with these results. A further analysis was finally conducted by selecting the top five most important variables [COL5A1, IL18 binding protein (IL18BP), SLC12A5, Midkine (MDK), and retinoid X receptor gamma (RXRG)] from the SVM model as predictive genes.

**Figure 9:**
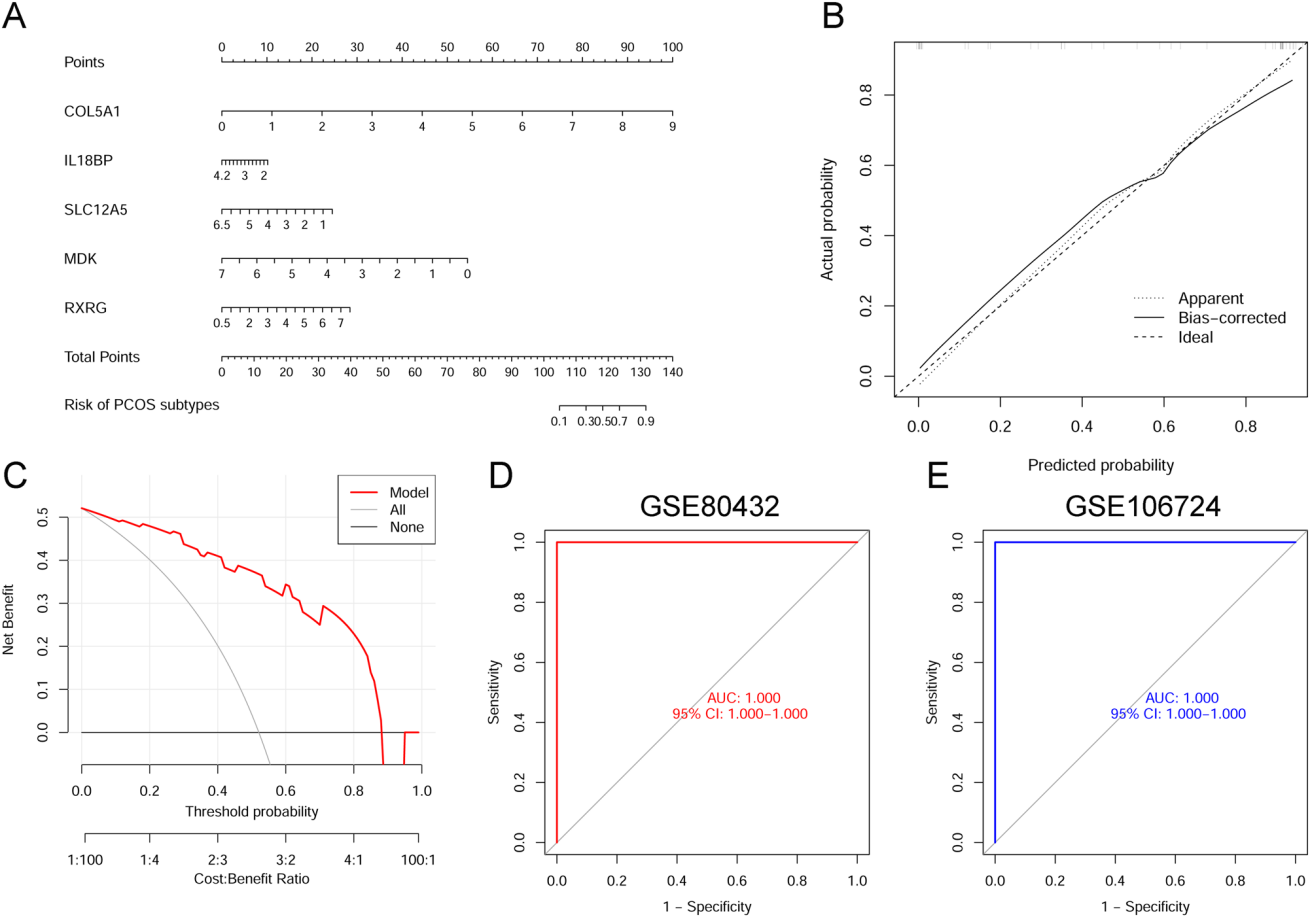
Verification of the 5-gene-based SVM model. (**A**) Construction of a nomogram for predicting the risk of PCOS clusters based on the 5-gene-based SVM model. (**B**,**C**) The prediction power of Nomogram was evaluated by the construction of calibration curve (**B**) and DCA (**C**). (**D**,**E**) ROC analysis of the 5-gene-based SVM model based on fivefold cross-validation in GSE80432 (**D**) and GSE106724 (**E**) datasets.

Our first step in evaluating the power of prediction in the SVM model was to construct a nomogram that could perform a risk assessment of cuproptosis clusters in 25 patients with PCOS (Fig. 9A). The calibration curve and DCA were used to assess the predictive accuracy of the nomogram model. The calibration curve results showed that the predicted and actual cluster risks of PCOS were very close (Fig. 9B), and as a result of DCA, we were able to demonstrate the high efficiency of our nomogram, which might contribute to clinical decision-making (Fig. 9C). After that, the other two datasets of ovarian granulosa cells including both healthy controls and patients with PCOS were used to validate our 5-gene prediction model. In both GSE80432 and GSE106724 datasets, the ROC curves displayed satisfactory performance with an AUC value of 1.000 (Fig. 9D,E). Hence, it was equally well in separating patients with PCOS from normal people when using our diagnostic model.

## Discussion

Given the limitations of current treatment methods for the metabolic symptoms of PCOS and its heterogeneity, new and more practical strategies are essential for controlling PCOS^3,36^. To gain a deeper understanding of PCOS and to provide new insight into the treatment of PCOS, more appropriate molecular clusters need to be identified. Lately, cuproptosis is a unique form of copper-induced cell death, primarily manifested by the accumulation of mitochondrial lipoylated proteins, which is intimately involved in the progress of disease^8,37^. However, it is unknown how cuproptosis works and what its effect is on various diseases. In consequence, this study aimed to elaborate on the specific role of CRGs in the phenotypic development of PCOS and microenvironment immune responses. Moreover, a few cluster-specific DEGs of cuproptosis-related molecular clusters were used to predict PCOS subtypes.

A systematic comparison of CRGs expression profiles in adipose tissue between patients with PCOS and healthy controls was presented in this research. The expression of CRGs in patients with PCOS was different from that in healthy controls, suggesting that CRGs play a critical role in the occurrence or development of PCOS. Following that, we calculated the correlation among CRGs to reveal whether they were related to PCOS. We found that several CRGs showed prominent synergistic or antagonistic effects, as demonstrated by CRGs interactions in patients with PCOS. Moreover, a difference was also noted in the proportion of immune cells between patients with PCOS and healthy controls. Patients with PCOS had higher infiltration degrees in activated mast cells, M0 macrophages, monocytes, activated memory CD4^+^ T cells, memory B cells, and so on, demonstrating the potential role of these immune cells in the pathogenesis of PCOS. Furthermore, based on the expression of CRGs, unsupervised cluster analysis was used to clarify the different regulation patterns related to cuproptosis in patients with PCOS, and two clusters that were associated with cuproptosis were identified. Moreover, PCA showed that PCOS classification had a prominent significance. Cluster-specific DEGs demonstrated that Cluster2 was principally enriched in the import of RNA into mitochondria, localization of proteins, and cellular polysaccharide catabolic. Conversely, the characteristics of Cluster1 were associated with immune-related pathways, such as production or combination of complement components and cytokines. This finding was approximately similar to that of previous research on the enrichment analysis of genes in PCOS^38^, suggesting that Cluster1 has a higher degree of immune infiltration because it correlates with immune-related pathways.

According to the expression profiles of cluster-specific DEGs, this study examined how four machine-learning models (RF, SVM, GLM, and XGB) performed in terms of prediction. Compared with the other three models, SVM had the highest efficacy of prediction in the testing set (AUC = 1.000); thus, machine learning based on SVM had a high advantage in predicting PCOS subtypes. Then, five important variables (COL5A1, IL18BP, SLC12A5, MDK, and RXRG) were chosen to create a 5-gene-based SVM model. Some studies hypothesized that COL5A1 is involved in the differentiation of ovarian granulosa cells; however, its mechanism has not been clarified, so the role of COL5A1 in PCOS is still controversial^39^. Gonadotropin-releasing hormone (GnRH) neurons, as the last common pathway for the central regulation of reproduction, have long been implicated in gamma-aminobutyric acid regulation. The functional changes of KCC2 (SLC12A5) might regulate the response of GnRH neurons to gamma-aminobutyric acid. Further research is required to clarify this point^40^. The change of GnRH led to the abnormal regulation function of the hypothalamus–pituitary–ovary axis, which might be one of the mechanisms causing PCOS. As a multifunctional cytokine, interleukin-18 (IL18) plays an important role in ovarian physiology function. Moreover, IL18 is associated with follicular development and atresia, as well as ovulation and steroidogenesis^41^. IL18BP is a secreted 40 kDa glycoprotein with high affinity to IL18. In vitro IL18 stimulated the proliferation of theca cells and steroidogenesis, and IL18BP could neutralize these effects. A novel way of treating PCOS is expected to be found via related research^42^. MDK, a heparin-binding growth factor involved in the development, reproduction, and repair, is associated with the pathogenesis of several diseases^43,44^. Moreover, MDK plays a crucial role in regulating oocyte performance during meiotic resumption^45^. One of the clinical features of PCOS is ovulation dysfunction or loss, suggesting that MDK is involved in the formation mechanism of PCOS. Previously, RXRG was associated with the age of menarche^46^. In consequence, it might be related to the ovulation dysfunction of PCOS; however, more research is required to support this.

The other two validation datasets (AUC = 1.000 and 1.000) confirmed the highly accurate prediction of the 5-gene-based SVM model, providing a new perspective on PCOS diagnosis. Furthermore, COL5A1, IL18BP, SLC12A5, MDK, and RXRG were used to create a nomogram model for diagnosing PCOS subtypes. The model was found to have a noteworthy prediction effect, indicating that the prediction model has clinical application value. In conclusion, the 5-gene-based SVM model provided suitable results in evaluating PCOS subtypes and future diagnosis.

There are some limitations to our study. First, data mining and analysis of formerly published datasets were used to support our results, which were based on public databases and computational algorithms. Therefore, conducting an additional experimental evaluation to further verify the expression of CRGs is important. Second, the number of analytical samples was limited and data were retrieved from different tissues or cells, which might increase the heterogeneity of the analysis. Furthermore, more patients with PCOS must be analyzed to determine whether clusters related to cuproptosis are accurate, and the possible interrelation between immune responses and CRGs requires further research. Finally, it would be important to have more detailed clinical features to verify the effectiveness of the prediction model.

## Conclusion

All in all, our study revealed that CRGs were associated with infiltrated immune cells. The results also clarified the prominent heterogeneity of immunization among patients with PCOS with diverse cuproptosis clusters. A 5-gene SVM model was selected as the optimum machine learning model for precisely evaluating PCOS subtypes and predicting PCOS. For the first time, a critical role of cuproptosis in PCOS was uncovered by our research, and molecular mechanisms contributing to PCOS heterogeneity were further clarified. The CRGs and immune cells of PCOS need to be further studied, and in this way, PCOS could be clinically diagnosed and feasibly treated with immunotherapy.

## Data Availability

On the GEO website (https://www.ncbi.nlm.nih.gov/geo/), the datasets supporting the conclusion of this article may be accessed by the following data accession numbers: GSE5090, GSE43264, GSE98421, GSE124226, GSE80432 and GSE106724.
